# Small RNA Sequencing Revealed that miR4415, a Legume-Specific miRNA, was Involved in the Cold Acclimation of *Ammopiptanthus nanus* by Targeting an L-Ascorbate Oxidase Gene and Regulating the Redox State of Apoplast

**DOI:** 10.3389/fgene.2022.870446

**Published:** 2022-04-04

**Authors:** Ming Zhu, Xue Wang, Yanqiu Zhou, Jinhua Tan, Yijun Zhou, Fei Gao

**Affiliations:** ^1^ Key Laboratory of Ecology and Environment in Minority Areas, National Ethnic Affairs Commission, Minzu University of China, Beijing, China; ^2^ Key Laboratory of Mass Spectrometry Imaging and Metabolomics, National Ethnic Affairs Commission, Minzu University of China, Beijing, China; ^3^ College of Life and Environmental Sciences, Minzu University of China, Beijing, China; ^4^ Beijing Center for Disease Prevention and Control, Beijing, China

**Keywords:** miRNA, miR4415, cold acclimation, *Ammopiptanthus nanus*, L-ascorbate oxidase

## Abstract

MicroRNAs (miRNAs) are small endogenous single-stranded RNAs that regulate plant growth, development, and environmental stress response posttranscriptionally. *Ammopiptanthus nanus*, a rare evergreen broad-leaved shrub in the temperate area of Central Asia, can tolerate freezing stress as low as –30 degrees centigrade in winter, and miRNA might be involved in the cold acclimation which enables *A. nanus* to obtain tolerance to freezing stress. Systematic identification and functional analysis of the miRNAs involved in the cold acclimation in *A. nanus* may promote understanding of the miRNA-mediated gene regulation network underlying cold acclimation. Here, based on small RNA and degradome sequencing, 256 miRNAs and 1,808 miRNA-target pairs were identified in *A. nanus*. A total of 39 cold-responsive miRNAs were identified, of which 29 were upregulated and ten were downregulated. These cold-responsive miRNAs may participate in the cold acclimation by regulating redox homeostasis (miR398, miR4415, and miR408), calcium signaling (miR5225 and miR5211), growth and development (miR159 and miR390), and small RNA–mediated gene silencing (miR168 and miR1515). We found that miR4415, a legume-specific miRNA, is involved in the cold acclimation of *A. nanus* by targeting an L-ascorbate oxidase gene and then regulating the redox state of the apoplast. Our study provides important data for understanding the regulatory role of miRNA in the cold acclimation of *A. nanus*.

## Introduction

Low temperature is a common unfavorable factor that negatively affects plant growth, development, and productivity and limits the geographical distribution of plant species (Khan et al., 2017; Ding et al., 2020). According to the temperature range, low-temperature stress can be divided into cold stress (0–10°C, chilling temperature) and freezing stress (<0°C, freezing temperature). Cold stress can directly affect the activity of various biological macromolecules, including various enzymes and bio-membranes, resulting in metabolic disorders, photosynthesis inhibition, and increased levels of reactive oxygen species (ROS) (Liu et al., 2018). Under freezing stress, ice crystals are first formed in the intercellular space or apoplast of plants. The ice crystals gradually increase, leading to severe cell dehydration and plasma membrane damage and rupture, further causing intracellular protein denaturation, precipitation of various biological molecules, disorder of cell metabolism, large accumulation of ROS, and even cell death (Bredow and Walker, 2017). Plants in temperate regions can gradually develop tolerance to freezing stress by exposure to low nonfreezing temperatures in autumn, a process known as cold acclimation. During the long evolutionary process, plants have evolved fine-tuned molecular regulatory mechanisms at transcriptional, posttranscriptional, translational, and posttranslational levels to cope with low-temperature conditions (Krutzfeldt and Stoffel, 2006; Liu et al., 2019). Recent studies have shown that in addition to signal transduction components such as mitogen-activated protein kinase (MAPK) pathway and transcription factors such as dehydration responsive element-binding (DREB) that play key roles in stress signal transduction and gene expression regulation and noncoding RNAs, such as microRNA (miRNA), also play essential regulatory roles in cold acclimation (Yu et al., 2019).

miRNAs are a class of endogenous single-stranded non-coding small RNAs (sRNAs) of about 18–24 nt. miRNAs play vital regulatory roles in plant growth and development and respond to environmental factors by negatively regulating protein-coding genes at the posttranscriptional level, thus regulating various biological processes (Song et al., 2019). It has been shown that miRNAs are involved throughout the development of reproductive organs, leaves, and stems and respond to high salinity, drought, and low-temperature stress (Ma et al., 2019). The application of high-throughput sequencing techniques to investigate the effects of cold stress on the expression of the conserved and nonconserved miRNAs in plants has been reported in *Arabidopsis thaliana* (Sunkar and Zhu, 2004), *Oryza sativa* (Lv et al., 2010), *Zea mays* (Yang et al., 2011), *Glycine max* (Xu et al., 2016), and *Triticum aestivum* (Wang et al., 2014). Several miRNAs were found to be involved in plant response to cold stress, including miR156, miR159, miR167, miR168, miR169, miR172, miR319, miR393, miR396, miR398, and miR408 (Megha et al., 2018), and further functional studies revealed the regulatory roles of cold-responsive miRNAs in cold stress response in plants. For example, it was demonstrated that miR156 enhanced cold stress tolerance in rice by targeting the expression of transcription factor genes such as *SQUAMOSA promoter binding protein-like* (*SPL*) (Zhou and Tang, 2019). Furthermore, miR528 decreased the expression of transcription factor *MYB30* by regulating an *F-box domain–containing protein gene* (*Os06g06050*), leading to enhanced cold stress tolerance in rice (Tang and Thompson, 2019).

*Ammopiptanthus nanus* is a desert shrub belonging to Ammopiptanthus, Leguminosae. This plant species is mainly distributed in the junction of the western Tianshan Mountains and Kunlun Mountains in Xinjiang, Wuqia county of Xinjiang Uygur Autonomous Region, and Uzbekistan. *A. nanus* originates in the Paleotropical region and is a tertiary relic that survived through Mediterranean retreat and climate aridity. *A. nanus* is of considerable scientific value in investigating the changes of paleogeography, paleoclimate, and paleoflora in Central Asia. In addition, *A. nanus* plays a vital role in the maintenance of the fragile ecosystems in arid areas in Central Asia. It can also be used as an appropriate plant species for windbreak, sand fixation, and greening in arid areas. *A. nanus* has evolved high tolerance to low temperature, drought, and other adverse conditions in temperate deserts. It is noteworthy that *A. nanus* is a rare, evergreen, broad-leaved woody plant in the desert area of Central Asia. *A. nanus* can tolerate low-temperature environments as low as –30°C. Thus, *A. nanus* was proposed to be a crucial material for analyzing the mechanism of low-temperature tolerance and screening candidate genes used for genetic engineering for improving low-temperature tolerance of economic plants (Gao et al., 2016; Cao et al., 2019; Cao et al., 2020; Zhang et al., 2021). However, system identification of miRNAs in *A. nanus* has not been reported, and how miRNAs respond to cold acclimation in *A. nanus* is not clear till now.

In the current study, we conducted genome-wide identification of the conserved and species-specific miRNAs in *A. nanus* based on deep sequencing and performed the expression profiling of miRNAs and their targets under cold acclimation. Several conserved and species-specific miRNAs and their targets were identified, and some miRNAs were demonstrated to play roles in cold acclimation of *A. nanus via* negatively regulating their targets. We further analyzed the biological functions of a lineage-specific miRNA, miR4415, in cold acclimation. Our results provided important data for understanding the miRNA-mediated gene regulation network underlying the cold acclimation in *A. nanus*.

## Materials and Methods

### Plant Materials and Cold Acclimation Treatment

Seeds of *A. nanus* were collected from Wuqia county (75°1′E, 39°43′N), Xinjiang Autonomous Region, China. After surface sterilization with 75% (v/v) ethanol and soaking in water for 48 h at room temperature, the seeds were planted in commercial pots containing a 3:1 (v/v) mixture of perlite and vermiculite. Seedlings were grown in a plant incubator with a photoperiod of 16 h, at 22–28°C and relative humidity of 35%. The seedlings were irrigated every 4 days with a solution of half-strength Hoagland. Eight weeks after germination, seedlings with similar heights were randomly divided into two groups. The first group of seedlings were kept in the plant incubator at 22–28°C for 7 days, and half of the plants were used as the control group (CK), and the other half were exposed to freezing stress treatment (–30°C) for 1 day and used as the freezing-stressed control group (SCK). The other group of seedlings were exposed to cold treatment (5°C/4°C, light/dark) for 7 days for cold acclimation, and half of the plants were used as the cold-acclimated group (CA), and the other half were exposed to freezing stress treatment (–30°C) for 1 day and used as the freezing-stressed cold-acclimated group (SCA). Leaf samples were harvested from each group and used for biochemical parameter measurement. Leaf samples from CK and CA were used for small RNA sequencing, and leaf samples from CK were used for degradome sequencing.

### Biochemical Parameter Measurement

Malondialdehyde (MDA) and relative electrolyte leakage (REL) were measured using the methods described by Yasar and Yang, respectively (Yang et al., 1996; Yasar et al., 2008). The activities of malate dehydrogenase (MDH) and ascorbate oxidase (AO) were measured using kits manufactured by Solarbio Science & Technology Co., Ltd. (Beijing, China). The content of ascorbate (ASA) and dehydroascorbate (DHA) was measured using kits manufactured by Solarbio Science & Technology Co., Ltd. (Beijing, China). Five independent plant samples were used (*n* = 5) for each biochemical parameter measurement.

### Small RNA and Degradome Sequencing

RNA samples were prepared using the TRIzol reagent. The concentration and purity of total RNA samples were assayed using Bioanalyzer 2100 and the RNA 6000 Nano LabChip Kit (Agilent, CA, United States) with a RIN value of >7.0. For small-RNA sequencing, approximately 1 μg of total RNA was used to construct a small-RNA library according to the protocol of the TruSeq Small RNA Sample Prep Kits (Illumina, San Diego, United States). Single-end sequencing (36 bp) was conducted on an Illumina Hiseq2500 at the LC Sciences (Hangzhou, China) by following the manufacturer’s instructions. Three independent biological replicates were set for each group, and a total of six high-throughput small RNA sequencing data were surveyed. The raw data of small RNA sequencing have been submitted to the NCBI SRA database with accession numbers SRR10906487, SRR10906486, SRR10906485, SRR10906484, SRR10906483, and SRR10906482.

For degradome sequencing, 150 ng of the poly (A) RNA sample was used as input RNA and annealed with biotinylated random primers. After RNA fragments were captured using biotinylated random primers, a 5ʹ adapter was ligated to the RNAs with monophosphate at the 5ʹ end. Next, reverse transcription and PCR were performed. Finally, the resulting library was single-end sequenced using an Illumina Hiseq2500 platform at the LC Sciences (Hangzhou, China) by following the vendor’s recommended protocol. The raw data of degradome sequencing have been submitted to the NCBI SRA database under the accession number SRR17711209.

### Identification of the Conserved and Species-Specific miRNAs

miRNAs were predicted using the ACGT101-miRprogram (version 4.2, LC Sciences), and the predicted miRNAs were then checked manually to remove false miRNAs. The genome sequence of *A. nanus* was downloaded from GigaBase (Gao et al., 2018). The conserved miRNAs and species-specific miRNAs were determined by aligning to the mature sequences of miRNAs from all green plants in the miRBase database (http://www.mirbase.org) (Kozomara et al., 2019). Species-specific miRNAs are miRNAs that are not aligned to the miRNAs deposited in miRBase (mismatches >4 nt) and meet the criteria of high-confidence miRNAs according to the latest miRNA identification protocol (Kozomara and Griffiths-Jones, 2014).

### miRNA Target Prediction and Degradome Analysis

The targets of miRNAs were predicted by using psRNATarget with the settings (maximum expectation: 4.0; length for complementary scoring: 20 bp; target accessibility–allowed maximum energy to unpair the target site: 25.0; ﬂanking length around target site for target accessibility analysis: 17 bp in upstream and 13 bp in downstream; range of central mismatch leading to translational inhibition: 9–11 nt) (Dai et al., 2018). CleaveLand 3.0 was used for analyzing sequencing data with default parameters (Addo-Quaye et al., 2009). CPC2.0 was used to evaluate the coding potential of miRNA targets (Kang et al., 2017).

### Differential Expression Analysis of miRNAs

The read counts from CK and CA were normalized to transcripts per million reads (TPM) (Dewey, 2010), and the differentially expressed miRNAs were determined by comparing the conserved or species-specific miRNA expression between two groups. Genes with a fold change ≥1.5 or ≤0.67 and a *p*-value <0.05 were identified as differentially expressed miRNAs. The heatmap was generated by using TBtools (Chen et al., 2020).

### qRT-PCR Analysis of miRNAs and Their Targets

RNA samples were prepared from the leaves using TRIzol reagent. qRT-PCR analysis of miRNAs and their targets were performed using the methods described previously (Abla et al., 2019). All primers were designed based on the sequences of selected miRNAs and their targets (Supplementary Table S1). The internal control genes are U6 snRNA (for miRNA) and 18S rRNA (for target gene). Three technical replicates were performed for each analyzed gene, and the expression levels of miRNAs and their target genes were calculated using the 2^−ΔΔCt^ method (Varkonyi-Gasic et al., 2007).

### Promoter Analysis

Plant promoter prediction software PlantCARE (http://intra.psb.ugent.be: 8080/PlantCARE) was used to predict the *cis*-acting elements involved in stress response or hormone response, and 1000 bp upstream sequences of the stem-loop precursors of each miRNA were used for promoter prediction.

### Identification, Multiple Sequence Alignment, and Phylogenetic Analysis of miR4415

To identify miR4415 and their precursors, mature sequences of know miR4415 were downloaded from miRBase and used to search for homologous sequences using the BLAST program, allowing four mismatches. Then, 400-bp flanking sequence was retrieved from reference sequences and folded using the online tool mfold to evaluate their secondary structure. Only those genomic loci with a stem-loop structure with an MFE value less than 30 kcal/mol and having the typical secondary structure for miRNA precursors were retained for subsequent analysis. All plant genomes in Phytozome v12 (https://genome.jgi.doe.gov/portal/PhytozomeV12) were downloaded and used for identifying miR4415. Multiple sequence alignment was performed using Clustal and visualized with Weblogo. The phylogenetic tree of the precursor sequences of miR4415 was built by MEGA X using the neighbor-joining (NJ) method.

### Dual-Luciferase Reporter Assay

Dual-luciferase reporter assay was performed according to the method for studying miRNA function (Clement et al., 2015). The precursor sequence of *A. nanus* miR4415 was ligated to the pGreen II 62 SK vector, and the coding sequence of L-AO was ligated to the pGreen II 0800-LUC vector. All primers were designed based on the sequences of Pre-miR4415, L-AO, pGreen II 62 SK, and pGreen II 0800-LUC vector (Supplementary Table S1). Extraction of *A. thaliana* protoplasts was performed as described by Yoo et al. (2007). The luciferase signals were detected using a Bio-Lite TM Luciferase Assay system kit manufactured from Vazyme Biotech Co., Ltd. (Nanjing, China). Five independent repeats were used for this assay.

### Subcellular Localization Analysis

Subcellular localization analysis of L-AO protein was performed as described in the literature (Yin et al., 2021). The coding sequence of the *L-AO* gene was ligated to pCAMBIA1303, and *L-AO* was transiently expressed in tobacco leaves for 48–72 h and imaged by using a confocal laser-scanning microscope (Leica TCS Sp8).

### Extraction of the Leaf Apoplast Fluid of *Ammopiptanthus nanus*

The leaf apoplast fluid of *A. nanus* was obtained using the infiltration–centrifugation methods (O'Leary et al., 2014). Water was used as the buffer, a 60-ml syringe was used for leaf infiltration, and the resulting leaf apoplast fluid was recovered by centrifugation for 10 min at 3,000 g.

## Results

### Cold Acclimation Enhances the Tolerance to Freezing Stress in *Ammopiptanthus nanus*

**FIGURE 1:**
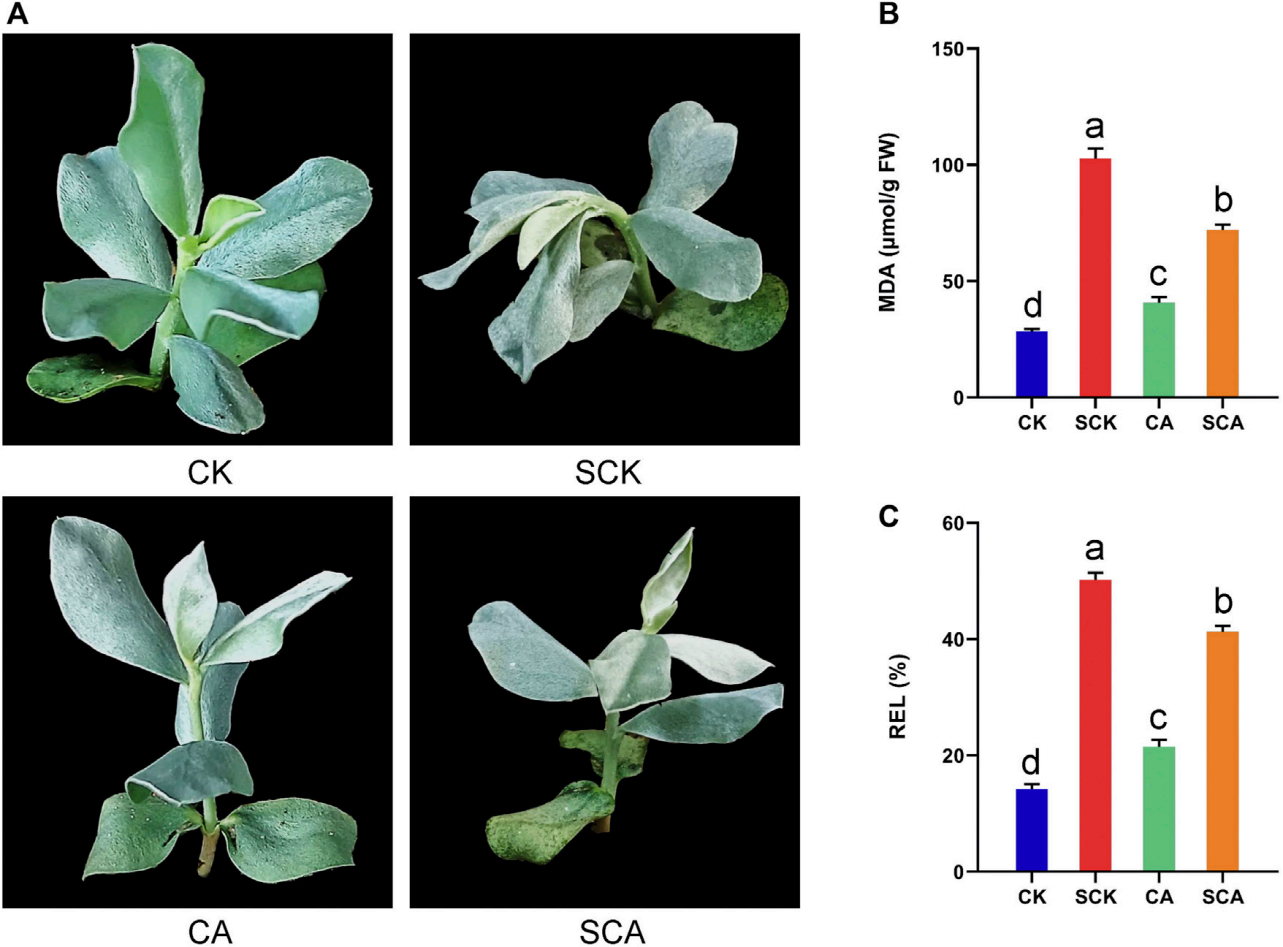
Cold acclimation enhanced the freezing stress tolerance of *A. nanus*. **(A)** Phenotype of *A. nanus* seedling in CK, SCK, CA, and SCA. The change of MDA **(B)** and REL **(C)** in the leaves of *A. nanus* before (CK and CA) and after freezing treatment (SCK and SCA). Values are expressed as means ± SD. Duncan’s method was used for multiple comparison analysis of variance, *n* = 3.

To evaluate the effect of cold acclimation on the tolerance to freezing stress in *A. nanus*, cold-acclimated and untreated *A. nanus* seedlings were subjected to freezing stress treatment, and MDA and REL, two biochemical indicators reflecting the degree of cell damage, were measured. The seedlings in SCK exhibited significant wilting, while the seedlings in SCA showed only slight wilting (Figure 1A). The MDA and REL values in SCA were significantly lower than those of SCK (Figures 1B,C), indicating that cold acclimation alleviated the damage to seedlings of *A. nanus* caused by the freezing stress treatment. In other words, high freezing tolerance can be obtained by cold acclimation in *A. nanus* seedlings.

### Cold Acclimation Resulted in a Significant Alteration in the Small RNA Population in *Ammopiptanthus nanus*

To evaluate the influence of cold acclimation on the sRNA population and identify miRNAs involved in cold acclimation in *A. nanus*, six sRNA libraries were constructed by using leaf samples isolated from CK and CA seedlings. CK and CA samples from *A. nanus* were sequenced by high-throughput sequencing technology. Approximately, 18 M raw sequence reads were obtained for each library. After removing adapters, low-quality sequences and sequences smaller than 18 nt, 12.28–16.10 M clean reads with sizes ranging from 18 to 25 nt were obtained for these sRNA libraries (Table 1).

**TABLE 1:** Statistics of the clean reads generated by high-throughput sequencing.

Library	Number of raw reads	Number of high-quality reads	Reads without 3′ adapter	Reads without insert fragment	5′ adapter contaminants	Reads with polyA	Reads smaller than 18nt	Number of clean reads
miCK_1	176,958,37 (100%)	173,638,56 (98.12%)	94,823 (0.54%)	158,912 (0.91%)	71,408 (0.41%)	728 (0.01%)	4,759,128 (27.40%)	12,278,857 (70.71%)
miCK_2	18,724,118 (100%)	18,316,541 (97.82%)	70,928 (0.38%)	147,893 (0.80%)	52,832 (0.28%)	1340 (0.01%)	3,316,221 (18.10%)	14,727,327 (80.40%)
miCK_3	19,377,347 (100%)	19,001,586 (98.06%)	61,387 (0.32%)	88,535 (0.46%)	59,142 (0.31%)	1097 (0.01%)	2,690,773 (14.16%)	16,100,652 (84.73%)
miCA_1	18,647,262 (100%)	18,277,927 (98.01%)	108,694 (0.59%)	128,250 (0.70%)	53,975 (0.29%)	918 (0.01%)	3,387,959 (18.53%)	14,598,131 (79.86%)
miCA_2	19,485,083 (100%)	19,126,024 (98.15%)	66,229 (0.34%)	109,935 (0.57%)	82,378 (0.43%)	576 (0.01%)	5,235,308 (27.37%)	13,631,598 (71.27%)
miCA_3	18,046,481 (100%)	17,705,703 (98.11%)	66,387 (0.37%)	130,810 (0.73%)	75,991 (0.42%)	504 (0.01%)	4,288,459 (24.22%)	13,143,552 (74.23%)

**FIGURE 2:**
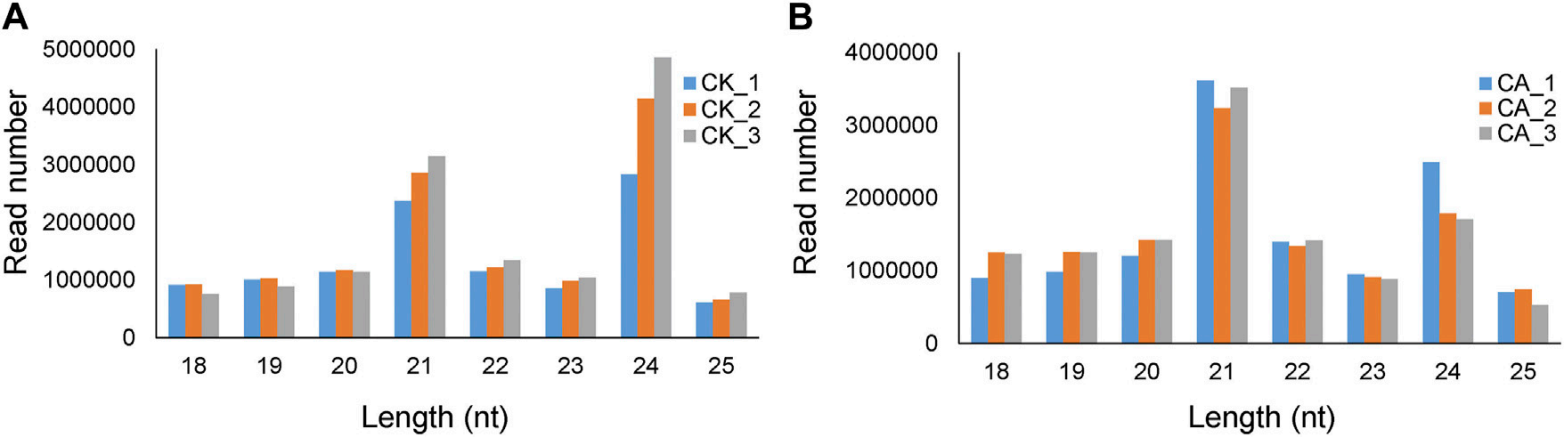
Length distribution of sRNA sequences in the six sRNA libraries prepared from leaves of the *A. nanus* seedlings cultured in untreated conditions (CK) **(A)** and under cold acclimation (CA) **(B)**.

The size distribution pattern of the three sRNA biological replicates in each group (CK or CA) was similar (Figure 2). In group CK, the top four abundant small RNA size categories were 24, 21, 22, and 20 nt groups, and such a size distribution pattern was similar to that of the typical plant mature sRNAs reported in several plant species, for example, *A. thaliana*, *Medicago truncatula* (Szittya et al., 2008), *O. sativa* (Morin et al., 2008), and *Citrus trifoliate* (Song et al., 2010). While there was a significant difference in the sRNA size distribution pattern between the CK and CA groups, the top two abundant sRNA size categories were 21 nt and 24 nt. These data suggested that cold acclimation resulted in a significant alteration in the sRNA population in *A. nanus* leaves.

### Identification of the Conserved and the Species-Specific miRNAs in *Ammopiptanthus nanus*

To identify miRNAs, all sRNAs of the six libraries were mapped to *A. nanus* genome sequences, and the flanking sequences were folded to determine whether a qualified hairpin could be formed. Consequently, 188 conserved mature miRNA sequences and 107 corresponding stem-loop precursors were identified (Supplementary Table S2). All the stem-loop precursors showed similarity to the miRNA precursors of green plants in the miRBase (Supplementary Table S2), indicating that these conserved miRNAs are generated from conserved stem-loop precursors. Most of the conserved miRNAs are supported by the simultaneous presence of the hairpin 5p and 3p arms, but no miR* sequences have been found for 80 stem-loop precursors. Among the 80 precursors, 35 with only 5p arm miRNAs and 45 with 3p arm miRNAs were found.

**FIGURE 3:**
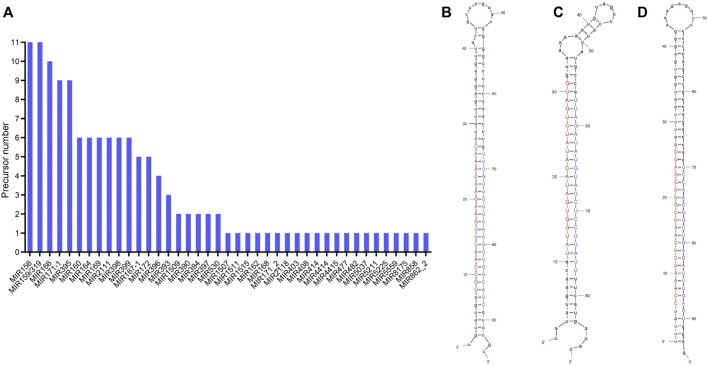
Statistics on the number of precursors of the miRNA families **(A)** and the secondary structure of the miRNA precursors of ana-miRN4 **(B)**, ana-miRN9 **(C)**, and ana-miRN33 **(D)**. Mature sequences of miRNAs are shown in uppercase, with red representing 5p and blue representing 3p. The structure diagrams were generated by using the UNAFold Web Server.

By aligning the stem-loop sequences obtained above with the miRBase database, 41 conserved miRNA families were identified (Figure 3A). MIR159 and MIR156, with up to 11 precursor loci, are the largest miRNA families, followed by MIR166 (10), MIR395 (9), and MIR171-1 (9). Twenty miRNA families, such as MIR162, MIR394, MIR477, MIR862-2, MIR1507, MIR1515, and MIR5559, had only one predicted precursor.

In the current study, all predicted miRNAs that have no homologs (with less than four mismatches) in miRBase were classified as species-specific miRNAs. According to the criteria for distinguishing the high- and low-confidence miRNA annotations (Kozomara and Griffiths-Jones, 2014), a total of 68 nonconserved miRNAs and 39 corresponding stem-loop precursors were identified with high confidence (Figures 3B–D; Table 2; Supplementary Tables S3).

**TABLE 2:** Species-specific miRNAs predicted from *A. nanus*.

ID	miRNA name	Sequence	Length (nt)	Total reads	ΔG (kcal/mol)
N1	ana-miRN1-5p	AAG​CTC​TTG​CTA​GGT​TGA​TTG	21	12	−70.20
	ana-miRN1-3p	ATT​GCC​TAC​AGT​TAG​ATC​CTT​A	22	281	
N2	ana-miRN2-5p	AGG​ATA​TTG​CTG​GGT​TGA​TTG	21	57	−76.80
	ana-miRN2-3p	ATT​ACC​TAT​AGT​TTT​ATC​CT	20	3	
N3	ana-miRN3-5p	TAC​TAA​TTT​GGT​CCT​CAA​GAA	21	11	−66.80
	ana-miRN3-3p	TCT​TGA​GGA​TCA​AAT​TTG​TAT​T	22	34	
N4	ana-miRN4-5p	TGC​CAA​CTT​GAA​GAA​CAA​ACA	21	150	−56.60
	ana-miRN4-3p	TTT​GTT​CTT​CAA​GTT​GGC​ATC	21	40,865	
N5	ana-miRN5-5p	TGT​TTG​GGA​GAA​GTG​GGA​AAG	21	243	−55.80
	ana-miRN5-3p	TTT​TCC​GCT​TCT​TTC​AAA​CAG	21	1791	
N6	ana-miRN6-5p	TCA​TTT​AAT​CCC​TAT​ACG​TAG	21	25	−38.80
	ana-miRN6-3p	TAA​CGG​GAG​GGA​CCA​AAT​GGG	21	101	
N7	ana-miRN7-5p	TAT​GGT​GAT​ACA​TAT​CTG​ATG	21	709	−36.10
	ana-miRN7-3p	TTA​GAT​ATG​TAT​CTC​CTT​ATT	21	772	
N8	ana-miRN8-5p	TCC​GTG​GCC​AAG​ATG​ACG​AAG​AA	23	1437	−40.70
	ana-miRN8-3p	CTC​TTC​ATC​TTC​CTC​GGA​TTC	21	43	
N9	ana-miRN9-5p	TTT​AGG​ATA​GGC​TTT​GAT​ACC	21	630	−54.30
	ana-miRN9-3p	TAT​CAA​AGC​CTA​TCC​GAG​ATC	21	16	
N10	ana-miRN10-5p	ATT​CTT​CAT​TTA​GTC​TCT​ATA	21	334	−40.70
	ana-miRN10-3p	TAG​GGA​CTA​AAT​GAA​GAA​TCT	21	10	
N11	ana-miRN11-5p	CAT​TAA​CGA​AAT​TCC​AAA​AAC	21	69	−93.40
	ana-miRN11-3p	TTT​TGG​AAT​TTC​GTT​AAT​ATC	21	393	
N12	ana-miRN12-5p	TCC​GCC​GAG​ACT​CGT​CTG​CA	20	172	−81.90
	ana-miRN12-3p	CAG​ACG​GGT​CAT​GGC​AGA​CGA	21	21	
N13	ana-miRN13-5p	TTT​CCT​GTC​ACA​TTC​TCT​GTC	21	49	−93.70
	ana-miRN13-3p	CAG​AGA​ATG​TGA​CAG​GAA​AGT​G	22	12	
N14	ana-miRN14-5p	TTG​TAC​GAT​TTT​GGT​CCC​TCA	21	12	−85.00
	ana-miRN14-3p	TGA​GGG​ACC​AAA​ATT​ACA​CAA​T	22	43	
N15	ana-miRN15-5p	TGG​GAT​TAC​AGG​GGT​TCT​CTT	21	10	−82.30
	ana-miRN15-3p	AGA​GAA​CCC​CTG​TAA​TCC​CAG	21	36	
N16	ana-miRN16-5p	TTA​CGG​GAC​ATT​CTT​ATG​TGG​C	22	14	−43.40
	ana-miRN16-3p	CAC​ATA​GGA​ATG​ACA​CGT​AAG​CT	23	20	
N17	ana-miRN17-5p	TTT​TGT​TTT​TGG​TCC​CTG​TCA	21	18	−54.00
	ana-miRN17-3p	CAG​GGA​CCA​AAA​ACA​AAA​TTT​T	22	10	
N18	ana-miRN18-5p	CCC​ACC​CAC​ACA​GTT​GAC​CTA​A	22	1057	−37.10
	ana-miRN18-3p	AGG​TCA​AAT​TTG​TGG​GGG​GTG	21	67	
N19	ana-miRN19-5p	GAG​GGA​CTA​AAT​GAA​GAA​TTT	21	105	−123.00
	ana-miRN19-3p	ATT​CTT​CAT​TTA​GTC​TCT​ATA	21	347	
N20	ana-miRN20-5p	TAA​GGA​CTA​AAT​GAA​GAA​TTT	21	32	−63.90
	ana-miRN20-3p	ATT​CTT​CAT​TTA​GTC​TCT​ATA	21	347	
N21	ana-miRN21-5p	CAT​TAA​CGA​AAT​TCC​AAA​AAC	21	67	−78.70
	ana-miRN21-3p	TTT​TGG​AAT​TTC​GTT​AAT​ATC	21	365	
N22	ana-miRN22-5p	CGT​CCT​GGT​GAA​ACG​CGC​CAC​T	22	307	−70.10
	ana-miRN22-3p	TGG​CGC​GTT​CCA​CCA​GGA​TGC	21	11	
N23	ana-miRN23-5p	TCC​ACT​GAA​CGT​AAT​TAA​CCA	21	227	−57.70
	ana-miRN23-3p	ATT​AAT​TAC​GTT​CAG​TGG​ATG	21	13	
N24	ana-miRN24-5p	TTG​ATC​AAC​TCC​TCC​ACC​GTG​A	22	25	−34.90
	ana-miRN24-3p	CAC​GGT​GAG​AGT​TGG​ACT​TTG​C	22	200	
N25	ana-miRN25-5p	TCC​ACT​GAA​CGT​AAT​TAA​CCA	21	210	−90.90
	ana-miRN25-3p	ATT​AAT​TAC​GTT​CAG​TGG​ATG	21	15	
N26	ana-miRN26-5p	TAC​CAA​TTT​GAT​CCT​CAA​GAA​T	22	10	−68.90
	ana-miRN26-3p	TTT​GAG​GAT​CAA​ATT​GGT​ATT	21	194	
N27	ana-miRN27-5p	TGG​CGC​GAC​GCA​GTG​GAA​GGC	21	72	−114.50
	ana-miRN27-3p	TCC​ACT​GCG​TCG​CGC​CAC​GTG	21	67	
N28	ana-miRN28-5p	TGG​CGC​GAC​GCA​GTG​GAA​GGC	21	43	−83.10
	ana-miRN28-3p	TCC​ACT​GCG​TCG​CGC​CAC​GTG	21	67	
N29	ana-miRN29-5p	TCA​CTC​ATC​GTT​GGA​TCA​ATC	21	75	−32.50
	ana-miRN29-3p	TTG​ATC​TAA​CGG​TGC​GTG​AAT	21	18	
N30	ana-miRN30-5p	TAG​CAC​ATC​ATG​TCC​CAA​TCA	21	12	−47.80
	ana-miRN30-3p	ATT​GGG​ATA​TAA​TGT​GAT​ACA​T	22	55	
N31	ana-miRN31-5p	CAT​TAA​CCA​CGA​TTT​TGA​ACG	21	37	−91.40
	ana-miRN31-3p	TTC​AAA​ATC​GTG​GTT​AAT​GAA	21	27	
N32	ana-miRN32-5p	TCC​GGA​TCC​TCT​AAC​TTT​AGG	21	49	−55.70
	ana-miRN32-3p	TAA​AGT​TAG​AGG​ATC​CAA​ATC	21	20	
N33	ana-miRN33-5p	CCA​AAT​CTC​AAT​CGT​TGG​ATT	21	39	−58.90
	ana-miRN33-3p	TCC​AAC​GGT​TGA​GAT​TTG​AGT	21	34	
N34	ana-miRN34-5p	CAT​TAA​CCA​CGA​TTT​TGA​ACG	21	37	−72.40
	ana-miRN34-3p	TTC​AAA​ATC​GTG​GTT​AAT​GAA	21	21	
N35	ana-miRN35-5p	TAT​CAA​TTT​GGT​CCT​CAA​GAA	21	32	−56.07
	ana-miRN35-3p	TCT​TGA​GGA​TCA​AAT​TTG​TAT​T	22	34	
N36	ana-miRN36-5p	TTT​GGA​TCC​TCT​AAC​TTT​AGG	21	15	−56.58
	ana-miRN36-3p	TAA​AGT​TAG​AGG​ATC​CGG​ATC	21	26	
N37	ana-miRN37-5p	TTT​GGA​TCC​TCT​AAC​TTT​AGG	21	15	−50.50
	ana-miRN37-3p	TAA​AGT​TAG​AGG​ATC​CGG​ATC	21	26	
N38	ana-miRN38-5p	TAT​CAA​TTT​GGT​CCT​CAA​GAA	21	32	−50.10
	ana-miRN38-3p	TTG​AGG​ACC​AAA​TTA​ATA​TTT​T	22	13	
N39	ana-miRN39-5p	CGG​AAA​ATT​GTT​GCA​GTT​AAG​C	22	41	−83.10
	ana-miRN39-3p	TTA​ACT​GCA​ACA​ATT​TGT​CCA​T	22	2655	

### Target Identification of miRNAs from *Ammopiptanthus nanus* Using Bioinformatics Analysis and Degradome Sequencing

To dissect the function of the predicted miRNAs in *A. nanus*, psRNAtarget, a miRNA target predicting tool, was used to identify miRNA target genes, and a total of 15,005 miRNA target pairs were predicted. Among them, 159 conserved miRNAs formed 10,159 miRNA-target pairs (Supplementary Table S4), and 64 nonconserved miRNAs formed 4,846 miRNA-target pairs (Supplementary Table S5).

**FIGURE 4:**
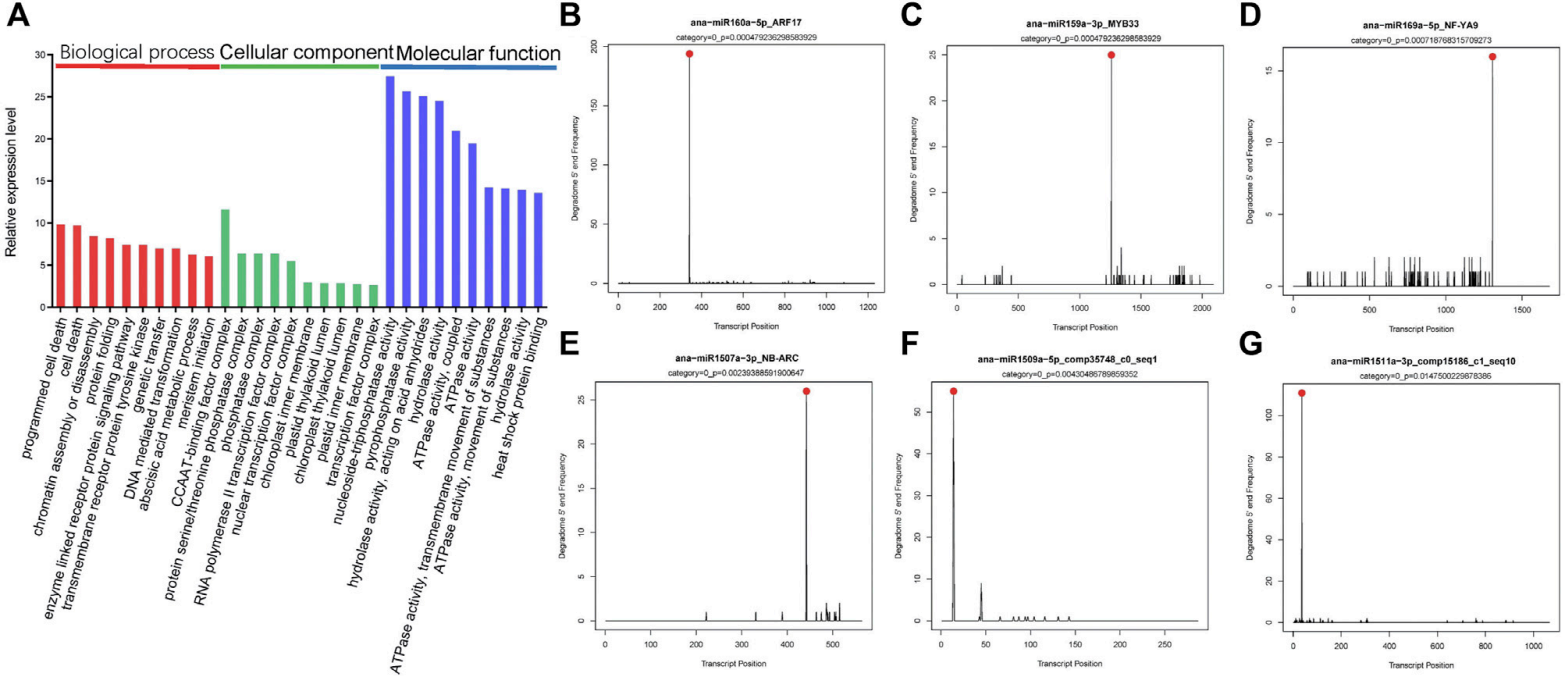
GO analysis and T-plots of miRNA targets in *A. nanus*. GO analysis of the miRNA targets **(A)**. Red columns represent BP, green columns represent CC, and blue columns represent MF. T-plots of miR160-*ARF* **(B)**, miR159-*MYB* **(C)**, miR169-*NF-YA* **(D)**, miR1507-*NB-ARC* **(E)**, miR1509-*comp35748_c0_seq1* **(F)**, and miR1511-*comp15186_c1_seq10* **(G)** pairs identified by degradome sequencing. Cleavage sites on target genes are indicated by red dots.

Through Gene Ontology (GO) functional classification analysis, the top 10 of the biological process (BP), cellular component (CC), and molecular function (MF) categories were obtained (Figure 4A). Among these groups, the term regulation of programmed cell death (GO: 0012501), cell death (GO: 0008219), CCAAT-binding factor complex (GO: 0016602), protein serine/threonine phosphatase complex (GO: 0008287), nucleoside triphosphatase activity (GO: 0017111), and pyrophosphatase activity (GO: 0016462) was the top two abundant terms in BP, CC, and MF categories. These results indicated miRNAs might be involved in pathways such as programmed cell death, chromatin assembly or disassembly, signal transduction, enzyme activity, transcription, and secondary metabolism in *A. nanus* by negatively regulating their target genes.

To experimentally identify the target genes of the predicted miRNAs in *A. nanus* in batch, degradome sequencing was conducted. High-throughput sequencing produced 11,048,980 reads representing the 5’ ends of uncapped, poly-adenylated RNAs. The total number of signatures matching the transcriptome sequences was 10,151,291 (91.88%), and the number of distinct sequences matching *A. nanus* transcriptome sequences was 2,440,623. CleaveLand pipeline was used to identify the cleaved miRNA targets in the *A. nanus* transcriptome. A total of 1,808 nonredundant putative splicing sites with *p*-values ≤1 were identified confidently (categories ≤ 4) (Supplementary Table S6). Among these predicted targets, 81 were classified in category 0, and they were the most credible targets. Of the 81 targets, 47 were known targets that were reported in other plant species previously, and 34 were noncanonical targets that were identified for the first time. Most of the 34 targets (21/34) were protein-coding genes, including *comp13662_c0_seq4* and *comp15069_c7_seq8*, which encode a global transcription factor group E8 and a basic-leucine zipper (bZIP) transcription factor family protein, respectively. Of the other 13 noncanonical targets, ten were predicted to be noncoding transcripts by CPC2.0, a coding potential calculator (Kang et al., 2017). (Figures 4B–G; Table 3).

**TABLE 3:** Noncanonical targets of the predicted miRNAs identified by degradome sequencing in *A. nanus*.

miRNA	Non-canonical target	Annotation/coding potential
ana-miR1507a-3p	comp13515_c0_seq1	Pre-rRNA-processing protein TSR2, conserved region
ana-miR1507a-3p	comp15069_c7_seq8	Basic-leucine zipper (bZIP) transcription factor family protein
ana-miR1507a-3p	comp19088_c0_seq1	TIM23-2 | translocase inner membrane subunit 23–2
ana-miR1509a-5p	comp15920_c0_seq10	hydrolases; protein serine/threonine phosphatases (PP)
ana-miR1509a-5p	comp15920_c0_seq1	hydrolases; protein serine/threonine phosphatases
ana-miR1511a-3p	comp15499_c1_seq10	Bromo-adjacent homology (BAH) domain-containing protein
ana-miR1511a-3p	comp15499_c1_seq2	Bromo-adjacent homology (BAH) domain-containing protein
ana-miR1511a-3p	comp15499_c1_seq9	Bromo-adjacent homology (BAH) domain-containing protein
ana-miR1511a-3p	comp15745_c1_seq1	GAUT6 | galacturonosyltransferase 6
ana-miR1511a-3p	comp19214_c0_seq1	ARM repeat superfamily protein
ana-miR1511a-3p	comp6070_c1_seq1	SWIB complex BAF60b domain-containing protein
ana-miR156d-3p	comp13662_c0_seq4	GTE8 | global transcription factor group E8
ana-miR156i-3p	comp15773_c2_seq28	DNA glycosylase superfamily protein (DGS)
ana-miR156j-5p	comp11270_c0_seq3	BPM2 | BTB-POZ and MATH domain 2
ana-miR156j-5p	comp16323_c0_seq1	*Glycine* cleavage T-protein family
ana-miR159h-3p	comp4558_c0_seq1	Mitochondrial transcription termination factor family protein
ana-miR159i-3p	comp14691_c0_seq1	Ypt/Rab-GAP domain of gyp1p superfamily protein (Gyp1p)
ana-miR171-1b-5p	comp15661_c0_seq18	Phox-associated domain, Phox-like, Sorting nexin, C- terminal (PX)
ana-miR171-1f-3p	comp14326_c1_seq1	Nucleotide/sugar transporter family protein
ana-miR396c-5p	comp11385_c0_seq1	LACS1 | AMP-dependent synthetase and ligase family protein
ana-miR396c-5p	comp19381_c0_seq1	BSD domain-containing protein
ana-miR1509a-5p	comp15155_c0_seq14	Noncoding
ana-miR1509a-5p	comp35748_c0_seq1	Noncoding
ana-miR1509b-5p	comp14487_c0_seq2	Noncoding
ana-miR1509b-5p	comp15155_c0_seq6	Noncoding
ana-miR1511a-3p	comp15186_c1_seq10	coding
ana-miR1511a-3p	comp15876_c0_seq40	Noncoding
ana-miR1511a-3p	comp390_c0_seq1	Noncoding
ana-miR156d-5p	comp12590_c1_seq4	coding
ana-miR156h-5p	comp12590_c1_seq2	coding
ana-miR164a-3p	comp14847_c0_seq4	Noncoding
ana-miR164c-3p	comp21396_c0_seq1	Noncoding
ana-miR396b-5p	comp10567_c1_seq1	Noncoding
ana-miR396b-5p	comp10567_c1_seq2	Noncoding

### Identification of the Cold Stress-Responsive miRNAs in *Ammopiptanthus nanus*

**FIGURE 5:**
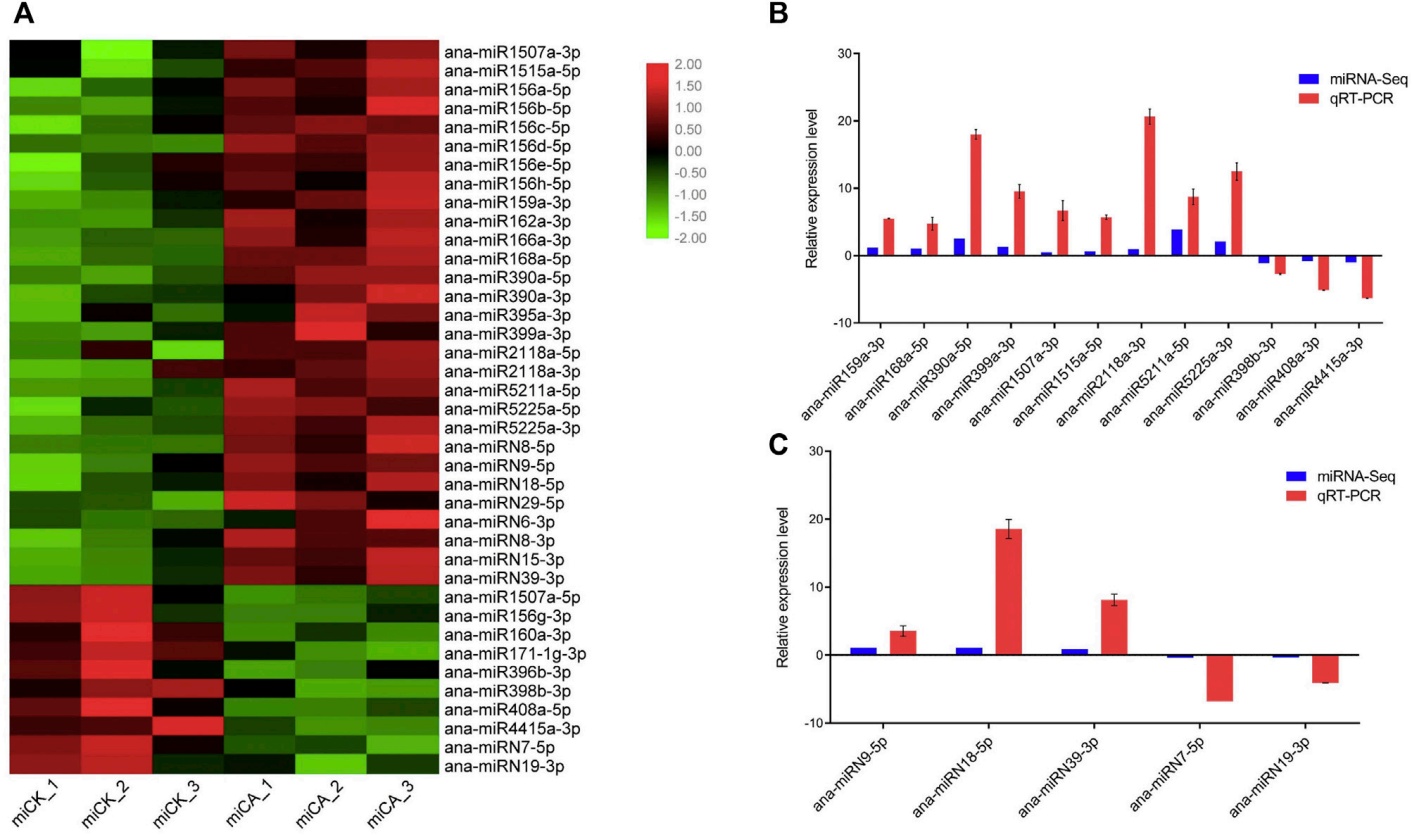
Expression of cold-responsive miRNAs in *A. nanus*. **(A)** Expression of cold-responsive conserved miRNAs and non-conserved miRNAs. **(B)** qRT-PCR validation of the cold-responsive conserved miRNAs. **(C)** qRT-PCR validation of the cold-responsive nonconserved miRNAs. The *Y*-axis shows the relative expression levels of conserved miRNAs and non-conserved miRNAs, with U6 as an internal reference, averaged over three technical replicates. Values are expressed as means ± SD (*n* = 3).

We analyzed miRNA expression by comparing the abundance of each miRNA in the group CK and CA base on the high-throughput miRNA sequencing (miRNA-seq) (Figure 5A), and 39 cold-responsive miRNAs were identified, including 29 conserved miRNAs and ten nonconserved. Twenty-one conserved miRNAs were upregulated under cold acclimation, including ana-miR156a-5p, ana-miR159a-3p, ana-miR162a-3p, ana-miR166a-3p, ana-miR168a-5p, ana-miR390a-5p, ana-miR395a-3p, ana-miR399a-3p, ana-miR1507a-3p, ana-miR1515a-5p, ana-miR2118a-3p, ana-miR5211a-5p, and ana-miR5225a-3p, and eight conserved miRNAs were downregulated, that is, ana-miR1507a-5p, ana-miR156g-3p, ana-miR160a-3p, ana-miR171-1g-3p, ana-miR396b-3p, ana-miR398b-3p, ana-miR408a-5p, and ana-miR4415a-3p. Among the differentially expressed nonconserved miRNAs, eight were upregulated under cold acclimation, that is, ana-miRN8-5p, ana-miRN9-5p, ana-miRN18-5p, ana-miRN29-5p, ana-miRN6-3p, ana-miRN8-3p, ana-miRN15-3p, and ana-miRN39-3p and two were downregulated, that is, ana-miRN7-5p and ana-miRN19-3p.

qRT-PCR experiments were conducted to validate the results of high-throughput sequencing. Twelve conserved miRNAs, including ana-miR159a-3p, ana-miR168a-5p, ana-miR390a-5p, ana-miR399a-3p, ana-miR1507a-3p, ana-miR1515a-5p, ana-miR2118a-3p, ana-miR5211a-5p, ana-miR5225a-3p, ana-miR398b-3p, ana-miR408a-3p, and ana-miR4415a-3p, and five nonconserved miRNAs, that is, ana-miRN9-5p, ana-miRN18-5p, and ana-miRN39-3p, ana-miRN7-5p, and ana-miRN19-3p were selected for qRT-PCR analysis (Figures 5B,C). The change patterns of *A. nanus* miRNAs revealed by qRT-PCR analysis were consistent with those calculated based on the miRNA-seq results.

### *Cis*-Elements Involved in Abiotic Stress Responses in the Promoters of the Cold-Responsive miRNA Genes

*Cis*-acting elements have been reported to be associated with plant responses to abiotic stresses, including cold stress (Zhang et al., 2005). To identify the *cis*-acting elements associated with the expression patterns of the cold-responsive miRNAs, we analyzed the 1000-bp upstream promoter sequence of the 12 cold-responsive miRNAs, which represented 12 cold-responsive miRNA families with higher abundance, by using a plant promoter prediction software, PlantCARE (http://intra.psb.ugent.be: 8080/PlantCARE For) (Lescot et al., 2002), and consequently, several stress response- and hormone response–related *cis*-acting elements, including the low-temperature responsive elements (LTRs), were found.

**FIGURE 6:**
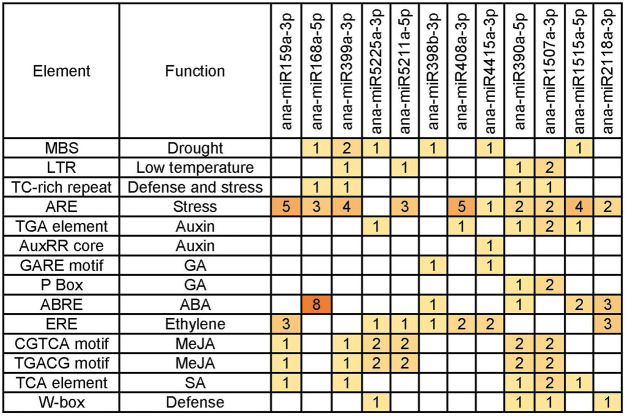
Abiotic stress–responsive c*is*-elements predicted from the promoters of cold-responsive miRNA genes.

Among the various *cis*-acting elements predicted from the promoters of the 12 cold-responsive miRNAs (Figure 6), the AU-rich element (ARE) was the most frequently detected *cis*-acting element, which was found in promoters of ten miRNAs, followed by ethylene response factor (ERE), appeared in promoters of seven miRNA genes. ARE has been shown to respond to hypoxic, low-temperature, and dehydration stress (Jang, 2016). Ethylene is an important gaseous phytohormone, playing a crucial role in plant growth and development and the response to biotic or abiotic stresses, and genes with ERE located in the promoter may be involved in ethylene response in plants (Rajesh et al., 2017). We also found several other *cis-*acting elements, including MBS, LTR, TC-rich repeat, TGA element, AuxRR core, GARE motif, P Box, ABRE, CGTCA motif, TGACG motif, TCA element, and W-box. ABREs were found in the promoter regions of most ABA-responsive genes and were reported to respond to abiotic stress in *A. thaliana* (Nagatoshi et al., 2018; Chang et al., 2019), and MBS is the MYB-binding site involved in drought inducibility (Lv et al., 2020).

### Expression Analysis of the Targets of the Cold-Responsive miRNAs in *Ammopiptanthus nanus*

**FIGURE 7:**
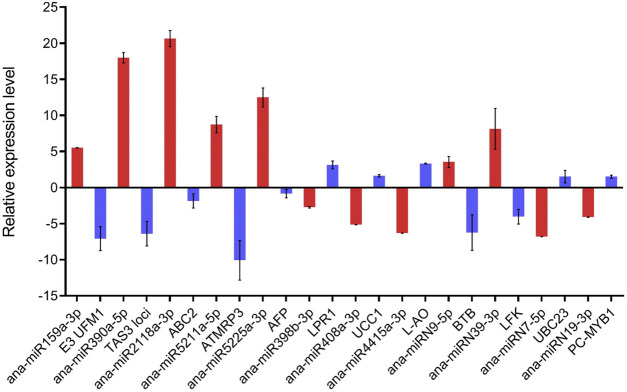
qRT-PCR analysis of the targets of the 12 selected cold-responsive miRNAs. Red columns represent miRNAs and blue columns represent target genes. The *Y*-axis shows the relative expression with U6 or actin as the internal reference, and data are averaged over three technical replicates. Values are expressed as means ± SD.

To investigate whether the expression of the predicted target negatively correlated with the expression of the corresponding miRNAs, we selected 12 predicted targets and analyzed their expression in cold-acclimated seedlings by qRT-PCR (Figure 7). After cold acclimation, seven miRNAs, including ana-miR159a-3p, ana-miR390a-5p, ana-miR2118a-3p, ana-miR5211a-5p, ana-miR5225a-3p, ana-miRN9-5p, and ana-miRN39-3p were upregulated, and the corresponding targets *E3 UFM1-protein ligase-like protein* (*E3 UFM1*), *TAS3*, *ABC2 homolog 13* (*ABC2*), *ATMRP3*, *Ankyrin repeat family protein* (*AFP*), *BTB-POZ and MATH domain 4* (*BTB*), and *LFK L-fructokinase* (*LFK*) were downregulated. The expression levels of ana-miR398b-3p, ana-miR408a-3p, ana-miR4415a-3p, ana-miRN7-5p, and ana-miRN19-3p were downregulated, and the corresponding targets *LPR1 Cupredoxin superfamily protein* (*LPR1*), *UCC1 uclacyanin 1* (*UCC1*), *L-AO*, *UBC23 ubiquitin-conjugating enzyme 23* (*UBC23*), *PC-MYB1 homeodomain-like protein* (*PC-MYB1*), *auxin response factor 3* (*ARF3*), *TIR1-NBS-LRR* (*TIR1*), *TIR2-NBS-LRR* (*TIR2*), *cold shock domain protein 1* (*CSDP1*), and *DICER-LIKE2* (*DCL2*) were upregulated. The expression of these 12 selected targets displayed the opposite pattern to that of the corresponding miRNAs after cold acclimation.

### miR4415 is a Legume-Specific miRNA

To understand the roles of miRNAs in cold acclimation, miR4415 was selected for further functional study. In the miRBase database (miRBase 21), there are two mature sequences of miR4415, namely, gma-miR4415a and gma-miR4415b. The mature sequences of miR4415 and the corresponding precursor sequences from *A. mongolicus* and *Astragalus membranaceus* have been identified by small RNA sequencing in previous studies (Gao et al., 2018; Abla et al., 2019). miR4415 is absent in both *A. thaliana* and rice, and thus we speculated that miR4415 might be a lineage-specific miRNA. We used the BLAST program to compare the mature sequences of the abovementioned miR4415 with the genomic sequences of all plant species in Phytozome v12 to detect the mature sequences of miR4415 in other plant species. Consequently, 11 additional mature sequences of miR4415 (guide strand) that were not included in miRBase 21 were identified from nine plant species, that is, *Glycine soja*, *Trifolium pratense*, *Cicer arietinum*, *Cajanus cajan*, *Vigna unguiculata*, *Vigna angularis*, *Medicago truncatula*, *Trifolium subterraneum*, and *Vigna radiata*. It is worth noting that all miR4415 (guide strand) are located at 3p of the stem-loop precursors.

**FIGURE 8:**
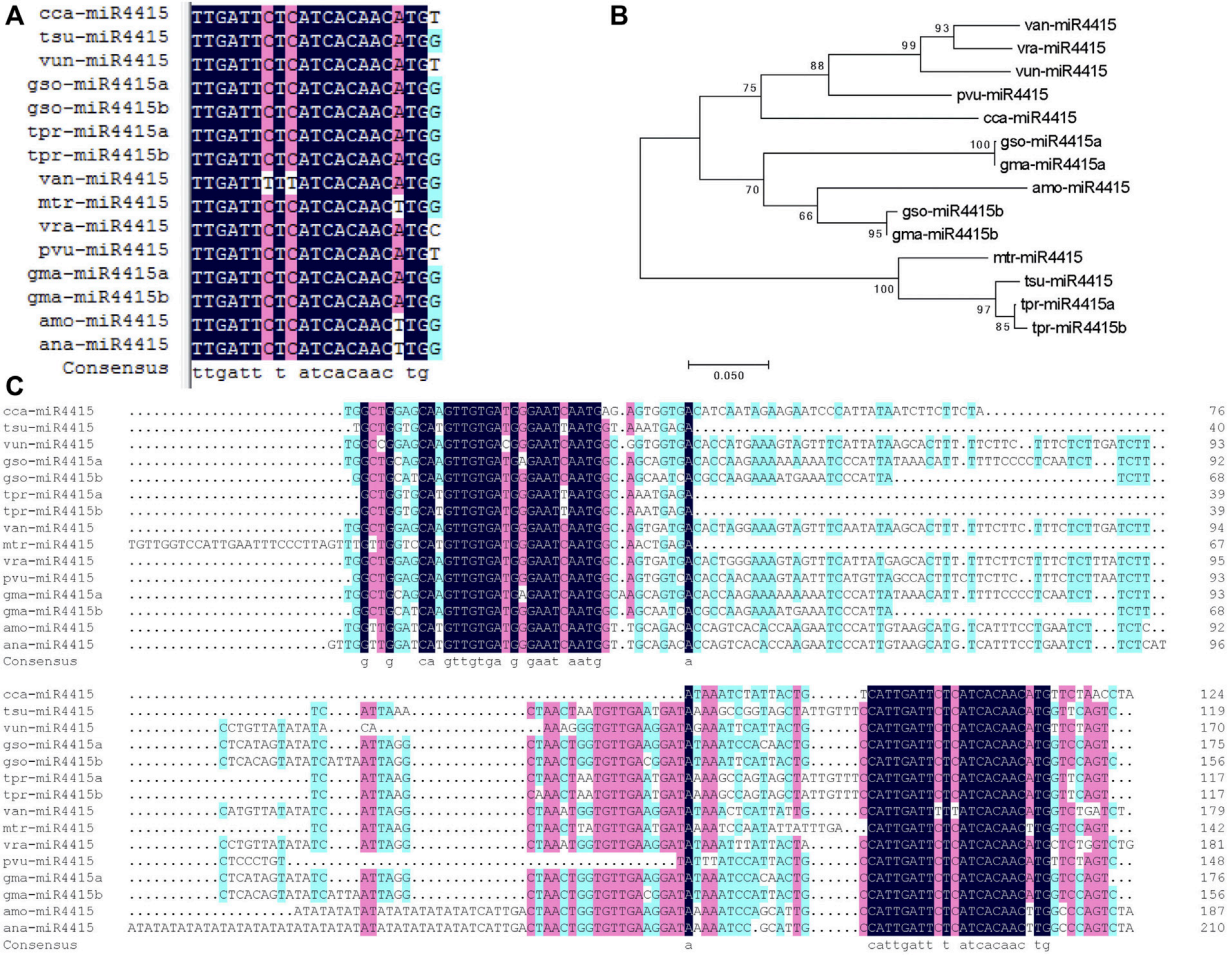
Bioinformatics analysis of miR4415 sequences. Multiple sequence alignment of the mature forms of miR4415 (guide strand) **(A)**. Phylogenetic tree of the precursors of miR4415 from several plant species **(B)**. Multiple sequence alignment of miR4415 precursors from several plant species **(C)**. cca-miR4415, miR4415 from *Cajanus cajan*; tsu-miR4415, miR4415 from *Trifolium subterraneum*; vun-miR4415, miR4415 from *Vigna unguiculata*; gso-miR4415a and gso-miR4415b, miR4415 from *Glycine soja*; tpr-miR4415a and tpr-miR4415b, miR4415 from *Trifolium pratense*; van-miR4415, miR4415 from *Vigna angularis*; mtr-miR4415, miR4415 from *Medicago truncatula*; vra-miR4415, miR4415 from *Vigna radiata*; pvu-miR4415, gma-miR4415a and gma-miR4415b, miR4415 from *Glycine max*; amo-miR4415, miR4415 from *Ammopiptanthus mongolicus*; ana-miR4415, miR4415 from *Ammopiptanthus nanus*.

Multiple sequence alignment analysis indicated that the mature forms of miR4415 from different species differed significantly at nucleotide 21, and minor differences were also found at nucleotide 7, 9, and 18 (Figure 8A). Multiple sequence alignment analysis of the precursor sequences revealed that in addition to the strong conservation of miR4415-3p and miR4415-5p, some nucleotides in other sites of the precursor also showed a high degree of conservation, such as the two nucleotides before the 5′ end of miR4415-3p (Figure 8C). These conserved nucleotides may play important roles in the formation of hairpin structures.

Phylogenetic analysis using the precursors of the 12 miR4415 indicated that all miR4415 were clustered into three clades. The first clade contained *van-miR4415, vra-miR4415, vun-miR4415, pvu-miR4415,* and *cca-miR4415*, the second contained *gso-miR4415a, gma-miR4415a, gso-miR4415b, gma-miR4415,* and *amo-miR4415*, while *mtr-miR4415, tsu-miR4415, tpr-miR4415a,* and *tpr-miR4415b* were clustered into the third branch. *Gma-miR4415a* and *gso-miR4415a* in soybean and wild soybean were clustered into one clade, while *gma-miR4415b* and *gso-miR4415b* were clustered into another clade (Figure 8B). Thus, *miR4415a* and *miR4415b* were already present before the species differentiation in soybean and wild soybean.

### miR4415 Targets an L-Ascorbate Oxidase

**FIGURE 9:**
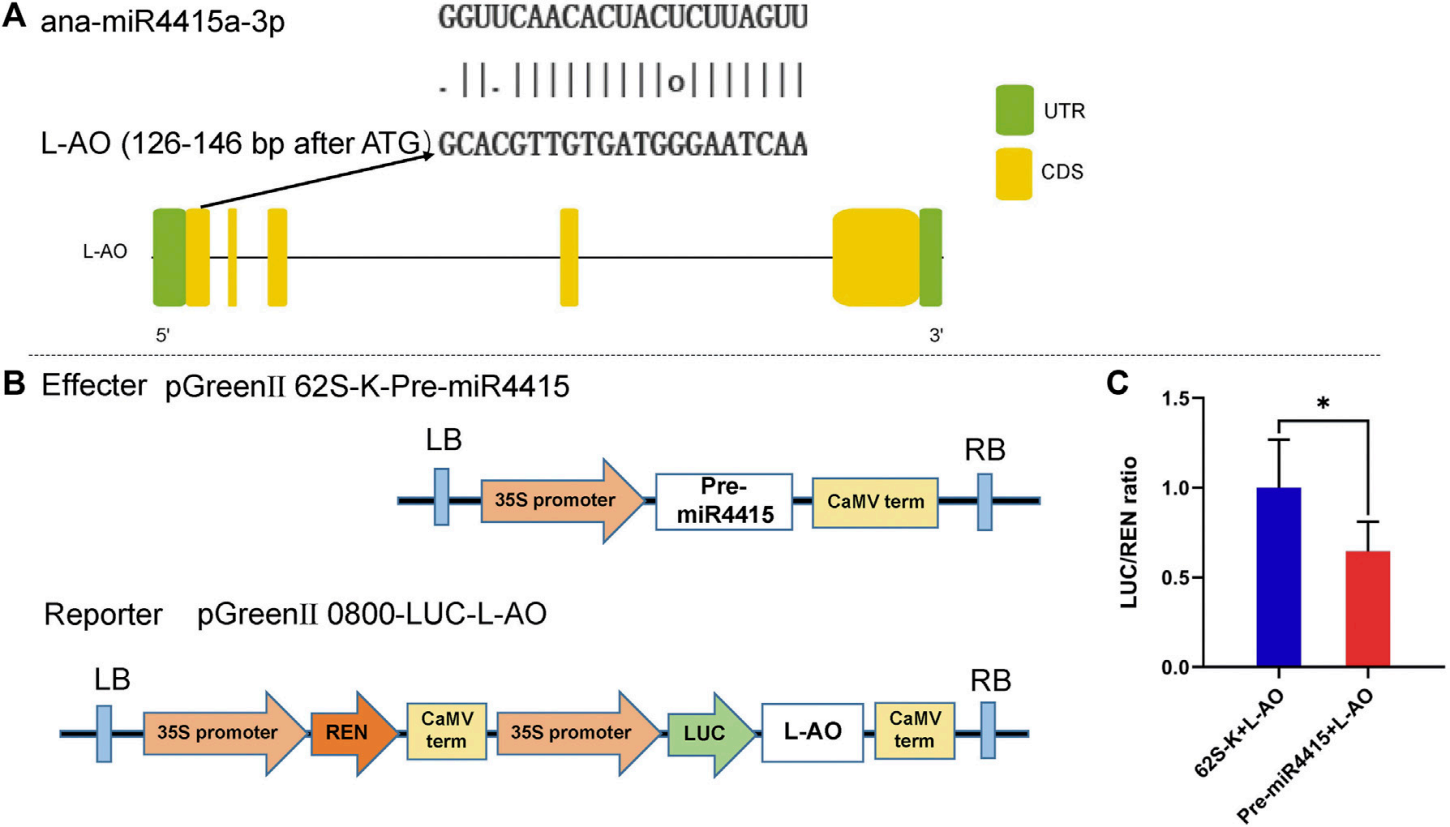
*L-AO* gene was targeted by ana-miR4415a-3p. Binding sites of ana-miR4415a-3p on *L-AO* as predicted by psRNAtarget **(A)**. Dual-luciferase reporter vector construction for dual-luciferase reporter assay **(B)**. Ratio of LUC/REN in the 62 SK + L-AO and Pre-miR4415 + L-AO cells **(C)**. Values are expressed as means ± SD (*n* = 5). Student’s t-test, *p < 0.05.

An *L-AO* gene (*EVM0030483.1*) was predicted to be the target of ana-miR4415a-3p by using the online software psRNAtarget (Figure 9A). Dual-luciferase reporter assay, a reliable method to detect the targeting relationship between miRNAs and their possible targets, was conducted to validate the prediction experimentally. Compared with 62 SK + L-AO, the Luciferase/Renilla (LUC/REN) ratio was significantly decreased in the Pre-miR4415 + L-AO cells (Figure 9B), indicating that miR4415 negatively regulated the expression of L-AO (Figure 9C). The opposite change pattern between ana-miR4415a-3p and *L-AO* (*EVM0030483.1*) in cold-acclimated *A. nanus* revealed by qRT-PCR analysis also supported the targeting relationship of miR4415 and *L-AO* (*EVM0030483.1*) (Figure 7).

### *Ammopiptanthus nanus* L-Ascorbate Oxidase is Located in the Apoplast

**FIGURE 10:**
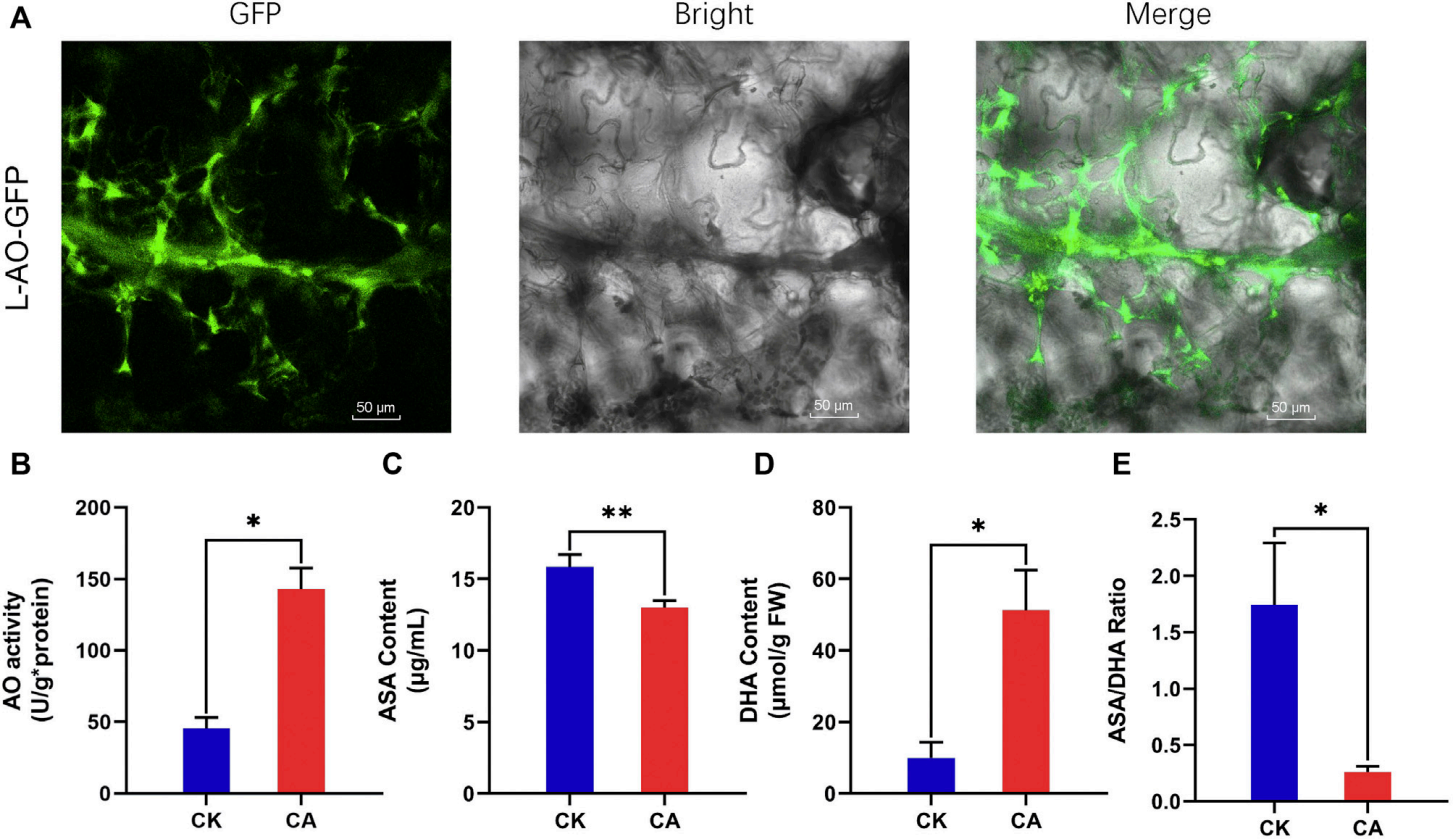
Subcellular localization of L-AO-GFP and analysis of apoplastic redox homeostasis in leaves of *A. nanus* under cold acclimation. **(A)** Subcellular localization of L-AO-GFP. Bars = 50 µm **(B)** AO activity. **(C)** Content of ASA. **(D)** Content of DHA. **(E)** ASA/DHA ratio. Values are expressed as means ± SD. Student’s t-test, *p < 0.05.

The L-AO (EVM0030483.1) protein was predicted to be located in the apoplast bioinformatically. To determine the subcellular localization of *A. nanus* L-AO (EVM0030483.1) experimentally, the *L-AO* (*EVM0030483.1*) gene was fused with the *GFP* gene and transiently expressed in *Nicotiana tabacum*. The GFP signal was mainly observed in the intercellular space, validating the apoplastic localization of L-AO protein (EVM0030483.1) (Figure 10A).

### L-AO Regulated the Contents of ASA and DHA in the Leaf Apoplast of *Ammopiptanthus nanus* Under Cold Acclimation

L-AO catalyzes the oxidation of ascorbic acid, which may affect the contents of ASA and DHA in the leaf apoplast of *A. nanus*. To assess the effect of upregulated L-AO on the concentration of ASA and DHA in the apoplast under cold acclimation, we extracted the apoplast fluid and measured the AO activity and ASA and DHA levels in the apoplast fluid of *A. nanus* before and after cold acclimation. Leaf extracts of *A. nanus* have MDH activity (4,755 u/g FW), while the MDH activity of the leaf apoplast liquid of *A. nanus* was not detected, indicating that the apoplast liquid we prepared had no cytoplasmic contamination and can be used for measurement of AO activity and the levels of ASA and DHA. The AO activity assay indicated that the AO activity in the apoplast of *A. nanus* was elevated after cold acclimation (Figure 10B). ASA and DHA measurements indicated a significant decrease in ASA (Figure 10C), a marked increase in DHA (Figure 10D), and a remarkable decrease in ASA/DHA ratio (Figure 10E) in the leaf apoplast liquid of *A. nanus* after cold acclimation.

## Discussion

*A. nanus* is a rare temperate evergreen broad-leaved plant, and it is of great significance to study its tolerance mechanism to freezing stress. As a class of endogenous regulatory RNA molecules, miRNAs not only participate in the regulation of plant growth and development but also in the response to environmental stresses (Song et al., 2019). Our study demonstrated that cold acclimation can improve the tolerance of *A. nanus* to the freezing stress, and miRNAs might play regulatory roles in cold acclimation in *A. nanus*. Analyzing the expression changes of miRNAs and their targets under cold acclimation is helpful in understanding the gene expression regulation network associated with the cold acclimation process. Our study systematically identified the miRNA and the corresponding targets that might be involved in cold acclimation and provided important data for understanding the molecular regulation mechanism of cold acclimation of *A. nanus*.

### The Conserved miRNAs Predicted in *Ammopiptanthus nanus*

The completion of genome sequencing of *A. nanus* makes it possible to systematically identify the miRNA in *A. nanus*. Most of the identified miRNAs in *A. nanus* were conserved miRNAs, and the precursor sequences of these miRNAs were distributed on different assembled genome contigs. Of them, several conserved miRNA loci were clustered in genome contigs (Supplementary Table S2). For example, three precursors of miR2111, that is, *ana-MIR2111a*, *ana-MIR2111b*, and *ana-MIR2111c* were clustered in Contig00573, and the other three miR2111 precursors, that is, *ana-MIR2111d*, *ana-MIR2111e*, and *ana-MIR2111f* were clustered in Contig00782. There are nine precursors in the MIR395 family, and seven of them were clustered in Contig00419, including *ana-MIR395a*, *ana-MIR395b*, *ana-MIR395c*, *ana-MIR395d*, *ana-MIR395e*, *ana-MIR395f*, and *ana-MIR395g*, and the other two precursors, that is, *ana-MIR395h* and *ana-MIR395i* were distributed closely in Contig00478. Some of these clustered miRNA loci are probably generated *via* gene duplication.

Based on their conservation among plant species, the 41 conserved miRNA families in *A. nanus* can be classified into four groups. Ten miRNA families, including MIR156, MIR159, MIR160, MIR166, MIR167_1, MIR 171, MIR390, MIR395, MIR396, and MIR408, showed a high level of conservation among multiple plant species and are presented in Bryophyta, Lycopodiophyta, Coniferophyta, and Magnoliophyta, but not in Chlorophyta. This group contains the most conserved miRNAs in the plant kingdom. Group 2 comprised 12 miRNA families, that is, MIR162, MIR164, MIR168, MIR169, MIR172, MIR393, MIR394, MIR397, MIR398, MIR399, MIR2111, and MIR2118. These miRNAs are mainly identified in Magnoliophyta and they represent classical higher plant miRNAs. miRNAs in Group 3 (MIR403, MIR858, MIR1509, MIR1511, and MIR5225) are present only in eudicotyledons; hence, they might originate before the emergence of eudicotyledons. The remaining miRNA families, including MIR1507, MIR1515, MIR4415, MIR5037, and MIR862_2, were identified only in legume plants such as *G. max* and *M. truncatula* and might be legume-specific miRNAs.

### The Species-Specific miRNAs Predicted in *Ammopiptanthus nanus*

Besides the 68 nonconserved miRNA, we also identified approximately 264 miRNA candidates in *A. nanus*. Although their putative precursors can be folded into qualified hairpins (Axtell and Meyers, 2018), the reads mapped to 5′ arm or 3′ arm of the hairpins were not found or the abundance of 5p or 3p in sRNA libraries is less than ten; thus, these sRNA cannot be annotated as miRNA with high confidence. Maybe increasing the sequencing depth will result in identifying more novel miRNAs.

The sequencing frequencies of the 68 identified species-specific miRNAs varied greatly (Supplementary Table S3). Although the expression levels of most of the species-specific miRNAs were very low, some of them were expressed in a considerable amount. More than one-third of the species-specific miRNAs (24/64, 35.29%) were sequenced more than 100 times, and five species-specific miRNAs (ana-miRN8-5p, ana-miRN18-5p, ana-miRN4-3p, ana-miRN39-3p, and ana-miRN5-3p) have read numbers of more than 1,000 (Supplementary Table S3). Among all the identified species-specific miRNAs, ana-miRN4-3p was the most abundant, with a read number of more than 40,000 (Supplementary Table S3). Some of the species-specific miRNAs with high expression levels might have developed under environmental pressure, and thus might be involved in the adaptive evolution of *A. nanus*.

### The Noncanonical Targets Predicted by Degradome Sequencing Analysis

Degradome sequencing has been proven to be an efficient method to identify plant miRNA target genes (Ma X. et al., 2015). In *A. nanus*, many miRNA-target pairs identified by degradome sequencing have been reported in other plant species, including *SQUAMOSA promoter binding protein-like (SPL)* genes as the targets of miR156, the *MYB* genes as the targets of miR159, *ARFs* as the targets of miR160, miR167, miR393, *endoribonuclease Dicer-like 1 (DCL1)* as the target of miR162, *nuclear factor Y genes (NF-Y)* as the target of miR169, *Integrase-type DNA-binding superfamily protein (AP2)* as the target of miR172, *ATP sulfurylase 1 (APS1)* as the target of miR395, *Laccase/Diphenol oxidase family protein (LAC)* as the target of miR397, *phosphate 2* as the target of miR399, *Argonaute family protein (AGO2)* as the target of miR403, *NB-ARC domain-containing disease resistance protein* as the targets of miR1507 and miR2118, and *autoinhibited Ca* ^*2+*^ *-ATPase* (*ACA*) as the target of miR5225. These results demonstrated the effectiveness of our degradome sequencing analysis.

Moreover, our degradome sequencing analysis also identified some noncanonical target genes of *A. nanus* miRNAs, which were not reported in other plant species. Among the 34 highly reliable target genes, which were classified in category 0, 21 had homologs in *A. thaliana* protein, and 13 cannot be annotated by the *A. thaliana* protein dataset, which were likely to be noncoding RNAs. Most of the noncanonical targets predicted in our study have no homology with the targets of the conserved miRNAs; thus, further investigations need to be conducted to understand the biological roles of these novel miRNA-target regulatory motifs.

### The miRNAs Were Involved in the Cold Acclimation in *Ammopiptanthus nanus*

**FIGURE 11:**
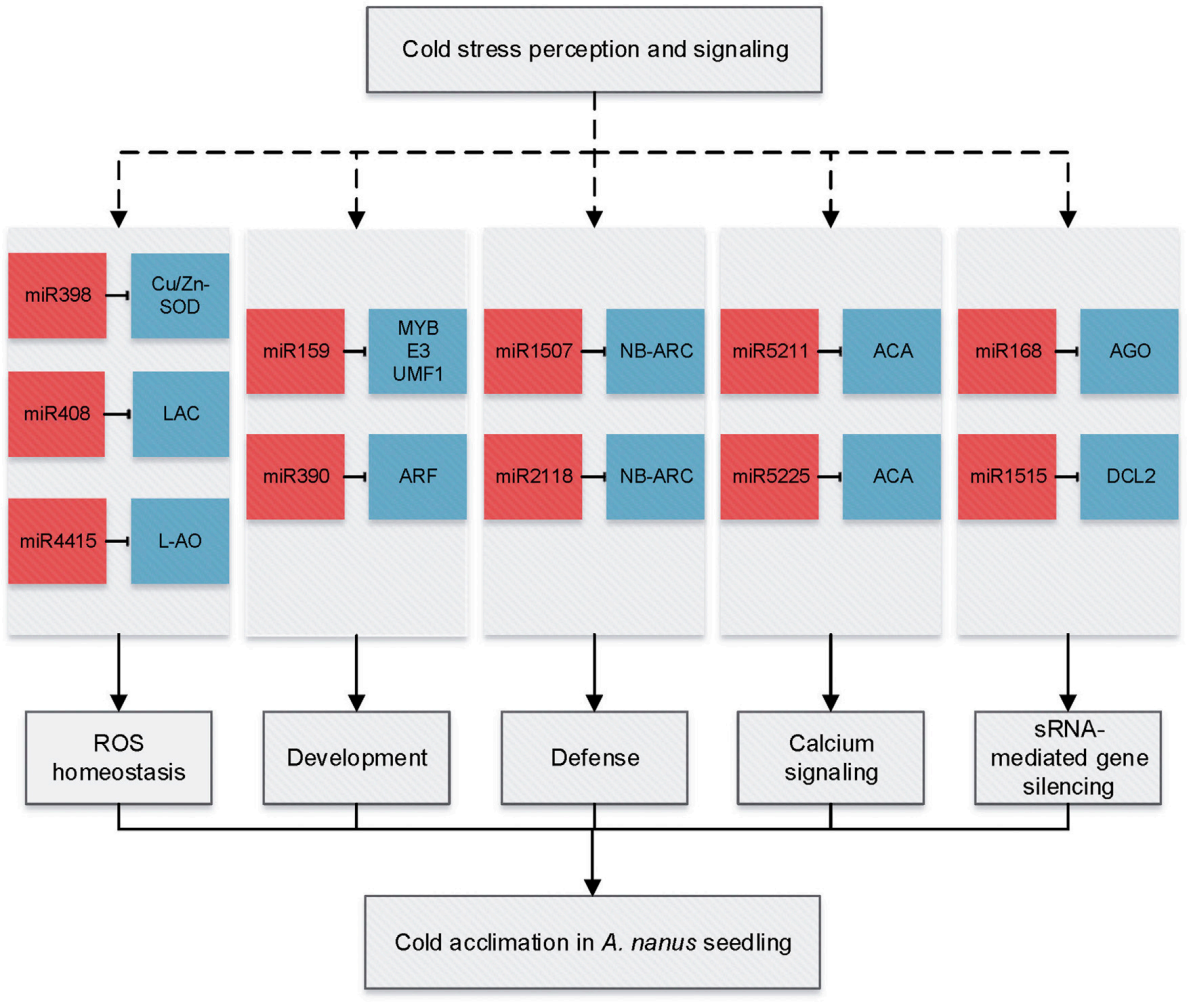
miRNA-mediated gene regulation network in response to cold acclimation in *A. nanus* leaves. miRNAs are indicated by red squares and targets by blue squares.

A total of 39 cold-responsive miRNAs were identified in *A. nanus*, including 29 conserved miRNAs and ten nonconserved. The 29 conserved miRNAs belonged to 20 miRNA families such as MIR159, MIR168, MIR399, MIR5211, MIR5225, MIR398, MIR408, and MIR4415. Twelve conserved miRNAs that represent most of these cold-responsive conserved miRNAs and five nonconserved miRNAs with higher abundance were further validated by qRT-PCR. These cold-responsive miRNAs might participate in cold acclimation by regulating multiple biological processes, including ROS homeostasis (miR398, miR408, and miR4415), development (miR159 and miR390), defense (miR2118 and miR1507), calcium signaling (miR5225 and miR5211), and small RNA (sRNA)–mediated gene silencing (miR168 and miR1515) (Figure 11).

Calcium is a ubiquitous signal and an essential macronutrient in plants, and calcium signaling plays a vital role in environmental stress signal transduction. Calcium ATPases catalyze the hydrolysis of ATP coupled with the translocation of calcium from the cytosol into the endoplasmic reticulum or out of the cell and are essential components of stress signaling. In *A. nanus*, miR5225 and miR5211 were predicted to target genes encoding calcium-transporting ATPases, and both were upregulated after cold acclimation, indicating that these miRNAs might be involved in cold acclimation by regulating the calcium signaling pathway.

Three miRNAs, that is, miR398, miR408, and miR4415 might be involved in cold acclimation by regulating their targets, *copper/zinc superoxide dismutase gene (CSD)*, *ceruloplasmin/laccase*, and *L-AO*, respectively. It is intriguing that all three targets encode multicopper oxidases (MCO). Multicopper oxidases are a class of enzymes that catalyze the oxidation of various substrates *via* the reduction of O_2_ to H_2_O without releasing activated oxygen species (Komori and Higuchi, 2015). Of them, ascorbate oxidase (AO, EC 1.10.3.3) is localized in the cell wall and widespread in the plant kingdom (Sanmartin et al., 2007). L-AO oxidizes AsA to DHA by monodehydroascorbate (MDHA) with the participation of O^2−^ and H_2_O_2_ while reducing oxygen to water, thereby regulating the redox state of the apoplast.

Laccases are classified as benzene diol oxygen reductases (EC 1.10.3.2) and are involved in lignin degradation and detoxification of lignin-derived product wound repair, disease resistance, stress resistance, and pigment synthesis in plants (Ranocha et al., 1999). Ceruloplasmin plays a vital role in copper metabolism and is also involved in scavenging anion radicals in plants (Bielli and Calabrese, 2002). miR408 was found to be downregulated in our study, which is consistent with previous reports in wheat and grape (Wang et al., 2014) but different from the observations in *A. thaliana* (Liu et al., 2008). Overexpression of miR408 promoted drought tolerance in chickpea (Hajyzadeh et al., 2015) and cold and oxidative stresses tolerance in *A. thaliana* (Ma C. et al., 2015).

CSDs are copper- and zinc-dependent enzymes that catalyze the conversion of superoxide radicals to H_2_O_2_, which can subsequently be reduced to H_2_O, and CSDs are proposed to play essential roles in scavenging the excessive ROS generated in plants under stressful conditions. Several reports have shown that miR398 plays an important role in plant antioxidant stress by regulating the expression of its target gene CSD (Jones Rhoades and Bartel, 2004; Sunkar and Zhu, 2004), although the expression pattern of miR398 was distinctly different. It has also been found that miR398 is upregulated in wheat but downregulated in rice when subjected to heat stress (Sailaja et al., 2014). Our data showed that miR398, miR408, and miR4415 were downregulated and their targets were upregulated in cold-acclimated *A. nanus* seedlings, and these miRNAs might contribute to cold acclimation by regulating ROS homeostasis in *A. nanus*.

miR159 has been reported to be induced by various abiotic or biotic stresses (Millar et al., 2019) and transient or mild upregulation of miR159 was observed in *A. thaliana* under cold stress (Liu et al., 2008). In *A. nanus*, we found that ana-miR159a-3p was upregulated under cold acclimation. The targets of ana-miR159a-3p in *A. nanus* encoded an MYB65 and an E3 UFM1-protein ligase–like protein (E3 UFM1). MYB TFs were involved in the regulation of various biological processes in plant growth and development in several plant species such as *A. thaliana* (Dubos et al., 2010; Chen et al., 2013) and rice (Yang et al., 2012). E3 ligase modulates plant abiotic stress responses through posttranslational modifications (Sun and Chen, 2004; Lyzenga and Stone, 2012). Ana-miR159a-3p may be involved in cold acclimation of *A. nanus* by negatively regulating *ATMYB65* and *E3 UFM1*.

### MiR4415 is involved in Cold Acclimation in *Ammopiptanthus nanus* by Regulating the Apoplastic Redox Status by Targeting an L-AO

Most miRNAs are conserved, to some extent, among different species, but not all miRNAs are highly conserved. Some miRNAs exist only in a specific evolutionary branch, and these miRNAs are called lineage-specific miRNAs. The lineage-specific miRNAs were evolved/developed in a specific evolutionary branch, hence contributing to developmental novelties during evolution (Zhang et al., 2008), and might be closely associated with some unique regulatory mechanism of the plants in this evolutionary branch. Our results demonstrated that miR4415 is a legume-specific miRNA family, which has only been reported in legumes such as *G. max* (Lei et al., 2019), *G. soja* (Zeng et al., 2012), *Cajanus cajan* (Nithin et al., 2017), *Phaseolus vulgaris* (Formey et al., 2015), *A. membranaceus* (Abla et al., 2019), and *A. mongolicus* (Gao et al., 2018).

miR4415 has been reported to be induced by aluminum treatment and drought stress but downregulated by biotic stress in *G. soja* (Zeng et al., 2012). miR4415 was observed to be downregulated by cold stress treatment in *A. nanus* and *A. membranaceus* (Abla et al., 2019) and by osmotic stress in *A. mongolicus* (Gao et al., 2018). These results indicated its involvement in the abiotic and biotic stress response.

AO was a key enzyme regulating the ROS status of the apoplast and an important regulatory enzyme for plant growth, development, environmental stress perception, and stress signal transduction (Foyer et al., 2020). AO can catalyze the oxidation of ASA to unstable MDHA and then form dehydroascorbic acid through nonenzymatic disproportionation. The redox status of the apoplast is largely determined by AO (Karpinska et al., 2018), and the change of ASA/DHA ratio caused by environmental stress may regulate gene expression and protein abundance through the change of redox state of the apoplast.

**FIGURE 12:**
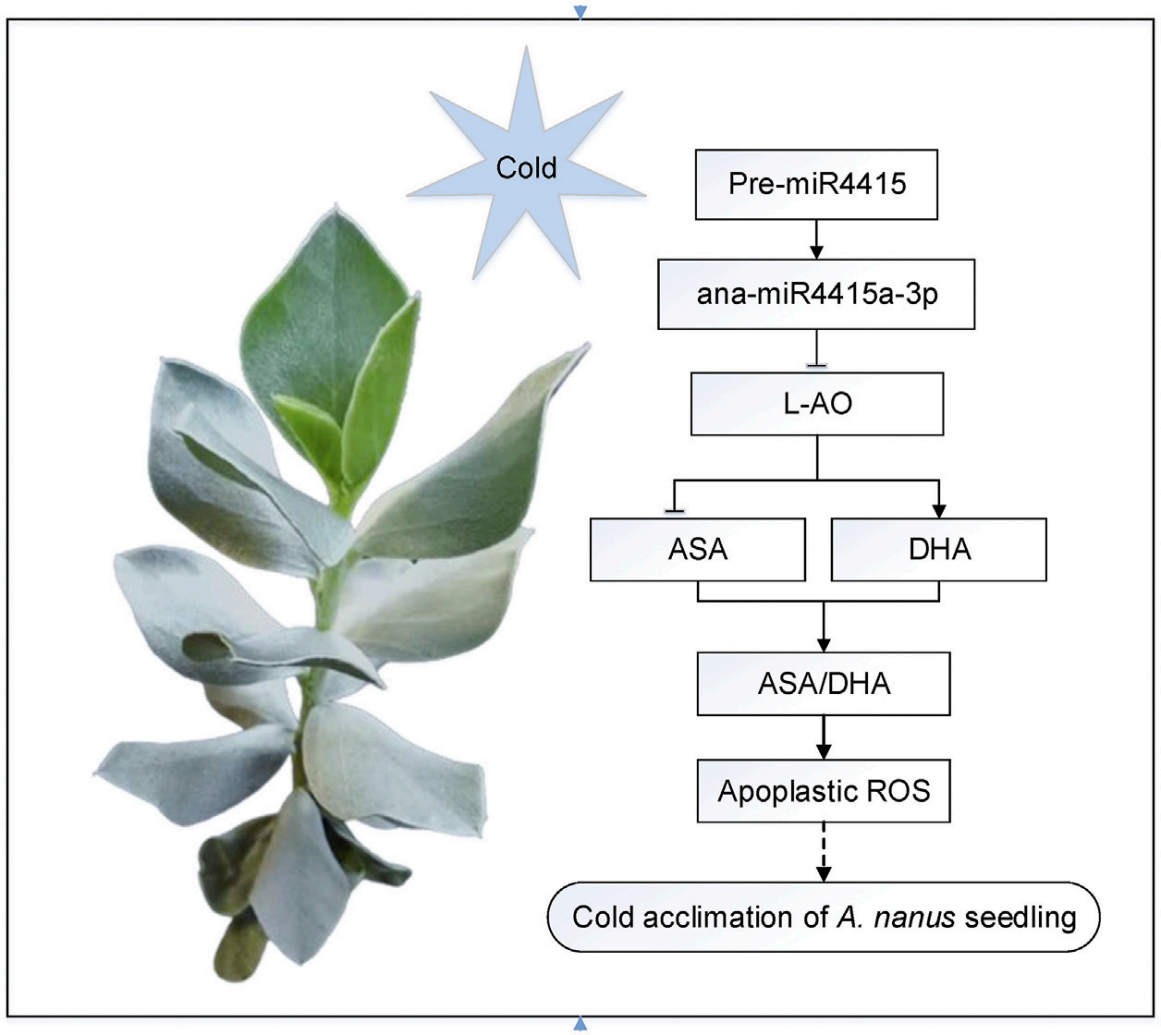
Proposed model of the regulatory role of miR4415 in cold acclimation in *A. nanus*. Under cold acclimation, expression of the miR4415 precursor was inhibited, resulting in downregulation of the ana-miR4415a-3p and upregulation of *L-AO*. Upregulated L-AO catalyzes the oxidation of ASA to form DHA, lowering the AsA/DHA ratio, thereby changing the redox status of the apoplast. Change in apoplastic redox state regulates the tolerance to freezing stress in *A. nanus* seedlings.

In *A. nanus*, expression of the miR4415 precursor was inhibited after cold acclimation, leading to downregulation of the ana-miR4415a-3p and upregulation of the *L-AO* gene. An elevated level of L-AO promotes the oxidation of ASA to form DHA and lowers the AsA/DHA ratio, resulting in alteration of the redox state of the apoplast. Change in the apoplastic redox state regulates the tolerance to freezing stress in *A. nanus* seedlings (Figure 12). A similar regulatory pathway has been reported in rice, in which miR528 regulated the apoplastic ROS state by targeting the *L-AO* gene, leading to enhanced tolerance to salt stress (Wang et al., 2021) and viral infection (Wu et al., 2017).

## Data Availability

The datasets presented in this study can be found in online repositories. The names of the repository/repositories and accession number(s) can be found below: https://www.ncbi.nlm.nih.gov/, SRR10906482https://www.ncbi.nlm.nih.gov/, SRR10906483https://www.ncbi.nlm.nih.gov/, SRR10906484https://www.ncbi.nlm.nih.gov/, SRR10906485https://www.ncbi.nlm.nih.gov/, SRR10906486https://www.ncbi.nlm.nih.gov/, SRR10906487https://www.ncbi.nlm.nih.gov/, SRR17711209.

## Supplementary Material

Supplementary Table S1 Primers used for qRT-PCR analysis.

Supplementary Table S2 Conserved miRNAs identified from *A. nanus*.

Supplementary Table S3 Species-specific miRNAs identified in *A. nanus*.

Supplementary Table S4 Targets of the conserved miRNAs predicted in *A. nanus* by psRNATarget.

Supplementary Table S5 Targets of the species-specific miRNAs predicted in *A. nanus* by psRNATarget.

Supplementary Table S6 Targets of the miRNAs identified in *A. nanus* by degradome analysis.
